# Application of a Novel Common-Iliac-Artery Skirt Technology (CST) in Treating Challenge Aorto-Iliac or Isolated Iliac Artery Aneurysms

**DOI:** 10.3389/fcvm.2021.745250

**Published:** 2021-10-18

**Authors:** Lunchang Wang, Chang Shu, Quanming Li, Ming Li, Hao He, Xin Li, Yin Shi, Jian Qiu, Tun Wang, Chenzi Yang, Mo Wang, Jiehua Li, Hui Wang, Likun Sun

**Affiliations:** ^1^Department of Vascular Surgery, The Second Xiangya Hospital of Central South University, Changsha, China; ^2^Vascular Disease Institute of Central South University, Changsha, China; ^3^Department of Vascular Surgery, Fuwai Hospital, Chinese Academy of Medical Sciences & Peking Union Medical College, Beijing, China; ^4^Department of Vascular Surgery, Fuwai Yunnan Cardiovascular Hospital, Kunming, China

**Keywords:** aorto-iliac artery aneurysm, iliac artery aneurysm, internal iliac artery, novel technology, endovascular, aortic surgery

## Abstract

**Purpose:** To report a novel common-iliac-artery skirt technology (CST) in treating challenge iliac artery aneurysms.

**Methods:** When required healthy landing zone of common iliac artery (CIA) is not available, CST is a strategy to exclude the internal iliac artery (IIA) and prevent IIA reflux without need of embolization. Patients who received endovascular aneurysm repair (EVAR) in our center from 2014 to 2020 were retrospectively screened, and patients treated with CST or with IIA embolization (IIAE) were enrolled.

**Results:** After retrospective screen of 524 EVAR patients, 39 CST patients, 26 IIAE patients, and 7 CST + IIAE patients were enrolled in this study. CST group suggested to have more aged, hyperlipemia, and smoking patients than IIAE group. Two groups had comparable maximal diameter of abdominal aorta (AA), CIA, EIA, but larger diameter of IIA (CST 19.82 ± 2.281 vs. IIAE 27.82 ± 3.401, *p* = 0.048), and CIA bifurcation (CST 25.01 ± 1.316 vs. IIAE 29.76 ± 2.775, *p* = 0.087) was found in IIAE group. Anatomy of 79.5% of CST patients and 92.3% of IIAE patients (*p* = 0.293) was not suitable for potential use of iliac branch device. CST group had significant shorter surgery time (CST 97.42 ± 3.891 vs. IIAE 141.0 ± 8.010, *p* < 0.001), shorter hospital stay (CST 15.35 ± 0.873 vs. IIAE 19.32 ± 1.067, *p* = 0.009), lower in-hospital [CST 0% (0/39) vs. IIAE 11.5% (3/26), *p* = 0.059] and 1-year follow-up stent related MAEs [CST 6.7% (2/30) vs. IIAE 28.6% (6/21), *p* = 0.052], but comparable mortality and stent related MAEs for all-cohort follow-up analysis comparing to IIAE group. In our study, a lower in-hospital buttock claudication (BC) rate for CST (CST 20.5% vs. IIAE 46.2%, *p* = 0.053) and a comparable erectile dysfunction (ED) rate (CST 10.3% vs. IIAE 23.1%, *p* = 0.352) were found between CST and IIAE groups. After 1 year, both groups had about one third relief of BC symptoms [CST 33.3% (4/12) vs. IIAE 30.7% (4/13), *p* = 1.000]. Subgroup analysis of 14 patents concomitant with IIA aneurysm in CST group and the 7 CST + IIAE patients were carried out, and no difference was found in mortality, stent MAEs, sac dilation, or reintervention rate. Last, illustration of seven typical CST cases was presented.

**Conclusion:** In selected cases, the CST is a safe, feasible-and-effective choose in treating challenge iliac artery aneurysms and preventing IIA endoleak.

## Introduction

Endovascular aneurysm repair (EVAR) has become the preferred way to treat abdominal aortic aneurysms (AAA) with suitable anatomy and life expectancy (1, 2). Iliac artery aneurysms (IAAs) are commonly coexisting with AAAs as aorto-iliac aneurysms in about 10–30% of AAA (3, 4), which poses significant clinical and technical challenges during EVAR (5, 6). Specifically, if the distal common iliac artery (CIA) does not present an adequate healthy landing zone, exclusion of internal iliac artery (IIA) or hypogastric artery is often required. Though benefits of preserving IIAs are now well-acknowledged and different strategies, devices, such as iliac branch device (IBD), are developed (7). Nevertheless, such techniques would significantly increase the surgery time and complexity and could be limited by anatomical constraints, technique experience, and availability of grafts. Remarkably, only 40.9% patients were suitable for use of iliac branch devices in an on-label fashion according to the manufacturer's instructions (8), and IBDs are not available in China until March 2021. And also, aneurysmal patients often complicated with cardiovascular diseases that require a feasible-and-effective procedure, particular for ruptured AAA patients (9, 10).

On the other hand, exclusion of the IIA is widely used and has been proven safe (11–15), and minimal adverse events of possible ischemic complications can be achieved as long as the contralateral IIA is fluent (14, 15). Coil or plug embolization of IIA, direct extension of graft to EIA, or combination of both is the main method for IIA exclusion. Extension of graft to EIA has risk of type II endoleak (16), while coil embolization is challenging and time-consuming with unfeasible anatomy. Here, we described a novel common-iliac-artery skirt technique (CST) in treating challenge aorto-iliac or isolated iliac artery aneurysms, which preserve the advantages of extension of graft to EIA and diminish risk of type II endoleak.

## Materials and Methods

### Enrollment and Data Collection

We retrospectively screened patients diagnosed with abdominal or iliac artery aneurysm and treated by endovascular procedures from 2014 to 2020 in our center. Among them, patients treated with CST or IIA embolization (IIAE) were enrolled in this study. Demographic data, risk factors and comorbidity, diagnosis, pre-procedure, procedure and in-hospital data, and follow-up results were analyzed. All patients received informed consent before the surgery, and this study was approved by the Institutional Review Board and the Medical Ethics Committee of our hospital.

A pre-surgical computed tomography angiogram (CTA) was used in all patients to assess extent of aneurysmal disease, tortuosity of the iliac vessels, and patency of the internal and external iliac arteries. Diameter of abdominal aortic and ipsilateral iliac arteries of IIAE or CST were measured from outer-wall to outer-wall of the long-axis. Length of CIA was retrospective collected by DSA with help of centimeter sizing pigtail catheter (Cook Medical Inc). Diameter and length were measured by two authors, and the average value of two authors' was used. Evaluation of erectile dysfunction was only carried out on male patients who are under 70s and had normal erectile function before surgery. Surgery time was calculated from anesthesia induction to the last frame of angiography. Two DSA rooms were routinly used by our group and due to the deficiency of one old-fashion DSA system; unfortunately, not all patients had radiation time and dosage data, so they were not analyzed in this study.

### Indications for CST or IIAE

CIAA without adequate healthy landing zone was the indication for CST or IIAE. Surgical method selection was based on general condition, comorbilities and vascular anatomy of each patient, and also patient's own will. In the IIAE group, embolization was performed preferentially *via* a contralateral approach before EVAR. IIAE was achieved using embolization coils (Cook Medical Inc) at the proximal site of the bifurcation of primary branches.

### Primary and Secondary End Points

Technical successful rate was defined as primary end points, and technique success including complete exclusion of aneurysms and no endoleak from IIA during surgery.

Secondary end points include surgery time, hospital stay, mortality, stent related major adverse events (MAEs), and reintervetion, while stent related MAEs include endoleak, limb occlusion, stent migration, contralateral IIA occlusion, and pelvic ischemia.

### Follow-Up and Statistics

Survival and reintervention events were followed by telephone calls directly with patients regularly. CTA imaging was carried out with available follow-up patients. Data are presented as median (range) or mean ± standard deviation. Data between two groups were analyzed using the Student *t* test, Chi-square, or Fisher's exact test analysis. A two-tailed *P* value of <0.05 indicated statistical significance.

## Results

### Procedure of a Classical Common-Iliac-Artery Skirt Technique (CST)

**Figure 1:**
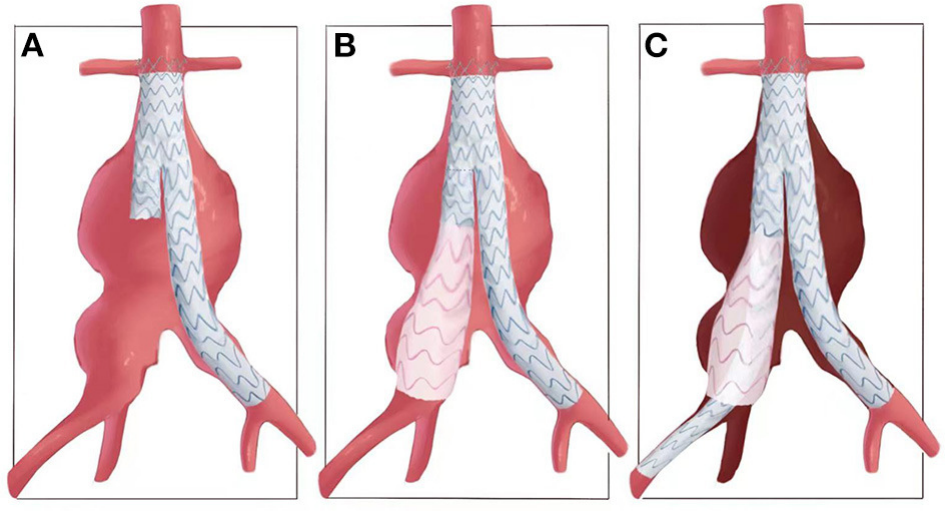
Illustration of common-iliac-artery skirt technology. **(A)** An aorto-iliac artery aneurysm and release of the bifurcated main body graft. **(B)** Then, a flared iliac limb is deployed at aneurysmal CIA and with its distal end locating at and sealing the iliac bifurcation, which is named as skirt-limb. **(C)** Another iliac limb with the same proximal diameter of skirt limb is deployed overlap with skirt-limb and elongated to EIA, with IIA excluded and possible reflux of IIA was restricted between the two iliac limbs.

As illustrated in Figure 1, after deployment of the aortic bifurcated grafts, with the shorter leg placed in aneurysmal CIA side, a flared iliac extension limb (skirt-limb) was deployed at aneurysmal CIA and with its distal end locating at and sealing the iliac bifurcation. The proximal end of skirt-limb with an identical diameter of the bifurcated shorter leg was placed within the leg, the diameter of distal end of skirt-limb should not smaller than the iliac bifurcation, and a 10% oversize of diameter was recommended but not obligatory.

And then, another iliac limb graft with the same proximal diameter of skirt limb was deployed overlap with skirt-limb and elongated to EIA, with IIA excluded and possible reflux of IIA restricted between two iliac limbs (Figure 1). Relationship of the two iliac stent grafts was similar with lady's skirt and leg, respectively.

For challenge iliac aneurysm, aortic extension configuration or cuff, or double CST skill can also be used in CST, which will be shown hereinafter.

### Demographics and Pre-Procedural Data

Retrospective screen found 524 patients received EVAR in our center from 2014 to 2020, and 39 CST patients, 26 IIAE patients, and 7 CST + IIAE patients were enrolled in this study. Given the small sample size of CST + IIAE group, regular statistical analysis only carried out between CST and IIAE group and most data of CST + IIAE group was not presented in the text, which but can be provided upon reasonable inquiry.

Demographic data, risk factors and comorbidity analysis suggested that CST group trend to have more aged, hyperlipidemia, and smoking patients than IIAE group, though not all data were statistically significant (Table 1). Male patients were overwhelming in both group, 97.4% for CST and 96.2% for IIAE group. Average age was 70.23 ± 0.969 for CST vs. 67.54 ± 1.383 for IIAE group (*p* = 0.105). Hypertension percentage was 66.7% for CST vs. 65.4% for IIAE (*p* = 1.000). CST group had a trend to have more patients with hyperlipidemia (CST 46.2% vs. IIAE 23.1%, *p* = 0.07), but two groups had no difference on coronary artery disease percentage (CST 20.5% vs. IIAE 23.1%, *p* = 1.000). CST group had a significant higher proportion of smoking patients (CST 94.8% vs. IIAE 61.5%, *p* = 0.002) and non-significant higher proportion of COPD patients (CST 23.1% vs. IIAE 11.5%, *p* = 0.334). Both groups had low prevalence of chronic kidney disease, while CST group had more patients with history of cerebral infarction (CST 15.4% vs. IIAE 3.85%, *p* = 0.228), though not significant.

**Table 1:** Demographics and preprocedural data.

	**CST**	**IIAE**	***p***
Male	97.4% (38/39)	96.2% (25/26)	1.000
Age	70.23 ± 0.969	67.54 ± 1.383	0.105
Hypertension	66.7% (26/39)	65.4% (17/26)	1.000
Hyperlipidemia	46.2% (18/39)	23.1% (6/26)	0.071
Smoking	94.8% (33/36)	61.5% (16/26)	0.002
CAD	20.5% (8/39)	23.1% (6/26)	1.000
COPD	23.1% (9/39)	11.5% (3/26)	0.334
CKD	2.56% (1/39)	0% (0/26)	1.000
History of CI	15.4% (6/39)	3.85% (1/26)	0.228
Diagnosis			0.253
AAA + CIAA	30.8% (12/39)	26.9% (7/26)	/
AAA + CIAA + IIAA	35.8% (14/39)	19.2% (5/26)	/
CIAA	15.4% (6/39)	15.4% (4/26)	/
CIAA + IIAA	18% (7/39)	38.5% (10/26)	/
**Diameter of (mm)**
AAA	40.25 ± 2.218	34.40 ± 3.203	0.129
CIA	41.22 ± 7.600	35.59 ± 2.893	0.585
CIA length	39.45 ± 3.676	33.79 ± 3.492	0.271
CIA_b_	25.01 ± 1.316	29.76 ± 2.775	0.087
IIA	19.82 ± 2.281	27.82 ± 3.401	0.048
EIA	9.951 ± 0.174	10.34 ± 0.290	0.231
Exclusion of IBD	79.5% (31/39)	92.3% (24/26)	0.293

*CAD, coronary artery disease; CKD, chronic kidney disease; CI, cerebral infarction; IBD, iliac branch device*.

Comparison of diagnosis and vascular anatomy confirmed that all enrolled patients were diagnosed with AAA concomitant with IAA or isolated IAA. Patients were divided into four categories based on diagnosis: AAA + CIAA, AAA + CIAA + IIAA, CIAA, CIAA + IIAA. No significant difference was found on proportion of diagnosis between CST and IIAE group (*p* = 0.253).

Two groups had comparable maximal diameter of AA (CST 40.25 ± 2.218 vs. IIAE 34.40 ± 3.203, *p* = 0.129), CIA (CST 41.22 ± 7.600 vs. IIAE 35.59 ± 2.893, *p* = 0.585), EIA (CST 9.95 ± 0.174 vs. IIAE 10.34 ± 0.290, *p* = 0.231) (Table 1), but larger diameter of IIA (CST 19.82 ± 2.281 vs. IIAE 27.82 ± 3.401, *p* = 0.048), and CIA bifurcation (CST 25.01 ± 1.316 vs. IIAE 29.76 ± 2.775, *p* = 0.087) was found in IIAE group. Based on the inclusion/exclusion criteria of the Gore Excluder Iliac Branch Endoprosthesis Trial and the Cook Zenith Iliac Branch Device Clinical Study (17, 18), enrolled patients in our study had a high exclusion rate for potential IBD use (CST 79.5% vs. IIAE 92.3%, *p* = 0.293).

### Procedure and In-Hospital Data

All surgeries were elective and performed under general anesthesia and by the same surgery team. Access was routinely achieved by cut-down femoral artery exposure. For primary end points, both groups had 100% technique successful rates and complete exclusion of aneurysms, though one case of IIAE group only achieved partial IIA embolization, but no endoleak from IIA was found after angiography (Table 2). For secondary end points, CST group had significant shorter surgery time (CST 97.42 ± 3.891 vs. IIAE 141.0 ± 8.010, *p* < 0.001) and shorter hospital stay (CST 15.35 ± 0.8734 vs. IIAE 19.32 ± 1.067, *p* = 0.009) compared to IIAE group. Both groups had low rate of Intensive Care Unit stay [CST 5.13% (2/39) vs. IIAE 7.69% (2/26), *p* = 1.000]. By analyzing skirt-limb's distal diameter, we found that 74.4% had a diameter of 24 mm, and 12.8% had a diameter of 28, including three 28–28 mm cuff. Preserve of contralateral IIA was guaranteed as long as suitable anatomy existed, and both groups had high rate of saving contralateral IIA (CST 92.3% vs. IIAE 76.9%, *p* = 0.140).

**Table 2:** Procedure and in-hospital data.

	**CST**	**IIAE**	***p***
**Primary end points**
Technique successful rates	100%	100%	1.000
**Secondary end points**
In hospital results
Surgery time (min)	97.42 ± 3.891	141.0 ± 8.010	<0.001
Hospital stay (day)	15.35 ± 0.873	19.32 ± 1.067	0.009
ICU stay rate	5.13% (2/39)	7.69% (2/26)	1.000
Distal diameter of Skirt stent (mm)	23.90 ± 0.344	/	/
20	12.8% (5/39)	/	/
24	74.4% (29/39)	/	/
28	12.8% (5/39)	/	/
28–28 cuff	7.69% (3/39)	/	/
Save of contralateral IIA	92.3% (36/39)	76.9% (20/26)	0.140
All-cause mortality	0% (0/39)	0% (0/26)	1.000
Stent related MAEs	0% (0/39)	11.5% (3/26)	0.059
Endoleak	0% (0/39)	0% (0/26)	1.000
Limb occlusion	0% (0/39)	3.8% (1/26)	0.400
Migration	0% (0/39)	0% (0/26)	1.000
Contralateral IIA occlusion	0% (0/39)	3.8% (1/26)	0.400
Ineffective IIAE	0% (0/39)	3.8% (1/26)	0.400
Reintervetion	0% (0/39)	3.8% (1/26)	0.400
Buttock claudication	20.5% (8/39)	46.2% (12/26)	0.053
Erectile dysfunction	10.3% (3/29)	23.1% (3/13)	0.352
Colonic ischemia	0% (0/39)	0% (0/26)	1.000
Pelvic necrosis	0% (0/39)	0% (0/26)	1.000

Analysis of in-hospital results found none mortality for both groups. CST had lower stent related major adverse events compared to IIAE group [CST 0% (0/39) vs. IIAE 11.5% (3/26), *p* = 0.059]. IIAE group had one limb occlusion, one unintended contralateral IIA occlusion, and one ineffective embolization of IIA. No endoleak or stent migration was found in both group. One reintervetion was applied in IIAE group due to limb occlusion. Both groups had pelvic ischemia complications, while CST had lower rate of buttock claudication (BC) (CST 20.5% vs. IIAE 46.2%, *p* = 0.053). Comparable erectile dysfunction (ED) (CST 10.3% vs. IIAE 23.1%, *p* = 0.352) was found, but no severe BC or ED require reintervention existed. No colonic ischemia and pelvic necrosis happened (Table 2).

### Follow-Up Results

CST group had 82.1% (32/39) and IIAE group had 92.3% (24/26) (*p* = 0.296) 1-year follow-up (Table 3). One-year all cause-mortality rate was 6.3% (2/32) for CST vs. 12.5% (3/24) for IIAE, *p* = 0.639. Stent MAEs increased in both groups after 1 year and IIAE group suggested having higher MAE events [CST 6.7% (2/30) vs. IIAE 28.6% (6/21), *p* = 0.052], which is consistent with in-hospital MAEs. In IIAE group, three limb occlusion and three contralateral IIA occlusion happened. In CST group, one limb occlusion and one contralateral IIA occlusion happened. No endoleak, stent migration, endotension or aneurysm sac dilation happened for both groups. Both groups only had one reintervetion [CST 3.3% (1/30) vs. IIAE 4.8% (1/21), *p* = 1.000] for symptomatic MAEs. After 1 year, both groups had about one third relief of BC symptoms [CST 33.3% (4/12) vs. IIAE 30.7% (4/13), *p* = 1.000], and IIAE had 33.3% (1/3) relief of erectile dysfunction.

**Table 3:** Follow-up results.

	**CST**	**IIAE**	***p***
**1-year results**
Follow-up rates	82.1% (32/39)	92.3% (24/26)	0.296
All-cause mortality	6.3% (2/32)	12.5% (3/24)	0.639
Stent related MAEs	6.7% (2/30)	28.6% (6/21)	0.052
Endoleak	0% (0/30)	0% (0/21)	1.000
Limb occlusion	3.3% (1/30)	14.3% (3/21)	0.293
Migration	0% (0/30)	0% (0/21)	1.000
Contral IIA occlusion	3.3% (1/30)	14.3% (3/21)	0.293
Endotension or Aneurysm sac dilation	0% (0/30)	0% (0/21)	1.000
Reintervetion	3.3% (1/30)	4.8% (1/21)	1.000
Relived buttock claudication	37.5% (3/8)	33.3% (4/12)	1.000
Relived erectile dysfunction	0% (0/3)	33.3% (1/3)	/
Colonic ischaemia	0% (0/30)	0% (0/21)	1.000
Pelvic necrosis	0% (0/30)	0% (0/21)	1.000
**All-corhot results**
Follow-up (month)	13–82 m	12–79 m	0.203
Median follow-up	35	26	0.203
All-cause mortality	9.38% (3/32)	12.5% (3/24)	0.679
Stent related MAEs	18.8% (6/32)	25% (6/24)	0.744
Endoleak	3.13% (1/32)	0% (0/24)	1.000
Limb occlusion	6.25% (2/32)	12.5% (3/24)	0.642
Migration	3.13% (1/32)	0% (0/24)	1.000
Other IIA thrombosis	6.25% (2/32)	12.5% (3/24)	0.642
Endotension or aneurysm sac dilation	3.13% (1/32)	0% (0/24)	1.000
Reintervetion	6.9% (2/32)	4.2% (1/24)	1.000

Analysis of all-cohort follow-up data was also carried out. The median follow-up month for CST was 35 (range 13–82), and 26 (range 12–79) for IIAE group (*p* = 0.203). No difference in mortality (CST 9.38% vs. IIAE 12.5%, *p* = 0.679), stent MAEs (CST 18.8% vs. IIAE 25%, *p* = 0.744), and reintervetion rate (CST 6.9% vs. IIAE 4.2%, *p* = 1.000) was found between two groups. CST group had one type II endoleak and sac enlargement of 5 mm from inferior mesenteric artery at 2-year follow-up, and regular follow-up was in process according to patient's willing (Table 3).

### Results of Different IIAA Treatment Strategies in CST Group

For patients diagnosed with IIAA, concerns may remain on risk of future IIAA enlargement by only covering the orifice of IIA. So subgroup analysis of 14 patents concomitant with IIA dilation in CST group without IIAE and the 7 CST+IIAE patients were performed (Table 4). The proportion of diagnosis was comparable. CST+IIAE group had larger diameter of CIA bifurcation (CST 29.01 ± 4.179 vs. CST+IIAE 42.40 ± 6.047, *p* = 0.082) and IIA (CST 35.31 ± 3.823 vs. CST+IIAE 48.47 ± 3.230, *p* = 0.038) and higher percentage of IIA > 35 mm [CST 42.9% (6/14) vs. CST+IIAE 100% (7/7), *p* = 0.018], and longer surgery time (CST 100.3 ± 5.781 vs. CST+IIAE 120.6 ± 6.962, *p* = 0.047). But all-cohort analysis with a median follow-up of 25.5 months for CST and 22.5 months for CST+IIAE (*p* = 0.296) showed no difference in mortality, stent MAEs, sac dilation, or reintervetion between two groups. Notably, both groups had one sac enlargement event. The one in CST group was described previously, and the sac enlargement in IIAAE group was thought to result from endotension, and open surgery was performed after 5-year follow-up (Table 4).

**Table 4:** Results of different IIAA treatment strategies in CST group.

	**CST**	**CST + IIAE**	***p***
Diagnosis			0.642
AAA + CIAA + IIAA	50% (7/14)	28.6% (2/7)	/
CIAA + IIAA	50% (7/14)	71.4% (5/7)	/
**Diameter of (mm)**
CIA	34.98 ± 4.175	46.14 ± 6.244	0.146
CIA_b_	29.01 ± 4.179	42.40 ± 6.047	0.082
IIA	35.31 ± 3.823	48.47 ± 3.230	0.038
IIA > 35 mm	42.9% (6/14)	100% (7/7)	0.018
Surgery time (min)	100.3 ± 5.781	120.6 ± 6.962	0.047
Save of IIA of other side	85.7% (12/14)	42.9% (3/7)	0.120
Loss of follow-up	21.4% (3/14)	14.3% (1/7)	1.000
Follow-up (month)	18–70 m	15–30 m	0.296
Median FU	25.5	22.5	0.296
All-cause mortality	9.1% (1/11)	16.7% (1/6)	1.000
Stent related MAEs	9.1% (1/11)	33.3% (2/6)	0.546
Endoleak	0% (0/11)	16.7% (1/6)	1.000
Limb occlusion	0% (0/11)	16.7% (1/6)	0.353
Migration	9.1% (1/11)	0% (0/6)	1.000
Endotension or Aneurysm sac dilation	0% (0/11)	16.7% (1/6)	0.353
Reintervetion	9.1% (1/11)	16.7% (1/6)	1.000

### Illustration of Typical and Challenge Cases With CST

**Figure 2:**
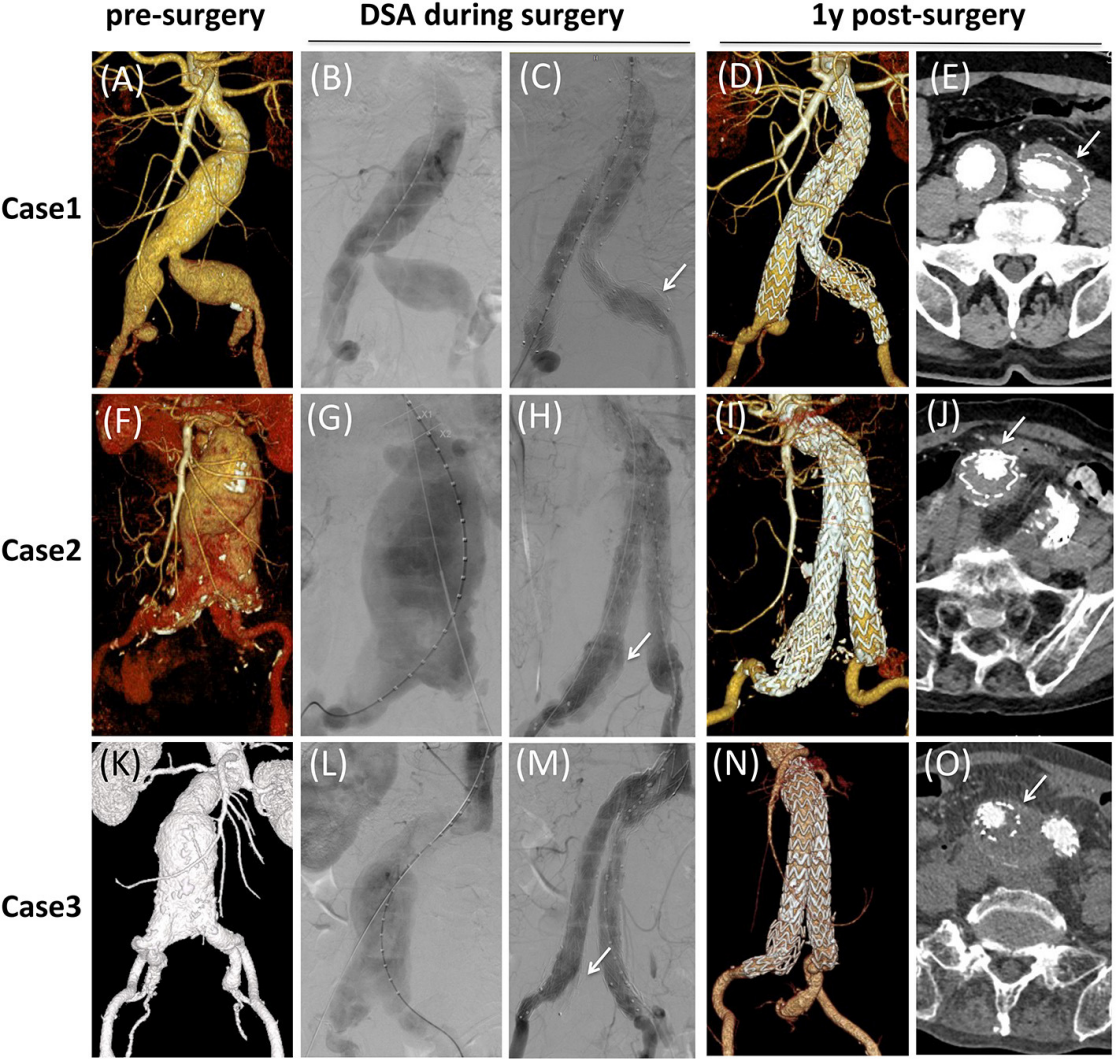
Typical cases of CST for aorto-iliac aneurysms. **(A,F,K)** Pre-surgical CTA 3D reconstruction. **(B,G,L)** DSA of aneurysms. **(C,H,M)** DSA after CST procedure. The white arrow indicates reflux of IIA was contained between the skirt graft and the extended iliac limb. **(D,I,N)** One-year follow-up CTA 3D reconstruction. **(E,J,O)** Transverse section of 1-year follow-up CTA. The white arrow indicates thrombosis formed in space between skirt graft and extended iliac limb.

To illustrate the application and advantage of CST, we presented several typical and challenge CST cases. Cases 1–3 showed three cases of AAA concomitant with a huge or short CIAA (Figure 2), and cases 1 and 3 were concomitant with IIAA at the same time. A Medtronic 16–28 mm flared skirt-limb placed in CIA and a 16–13 mm iliac limb extended to EIA was used to seal the CIAA and exclusion of one side IIA. From the DSA, we could notice that the reflux of IIA was contained between the skirt graft and the extended iliac limb (white arrow in Figures 2C,H,M). One year follow-up results all showed no endoleak or aneurysm sac dilation, and thrombosis formed in space between skirt-limb and extended iliac limb (white arrow in Figures 2E,J,O).

**Figure 3:**
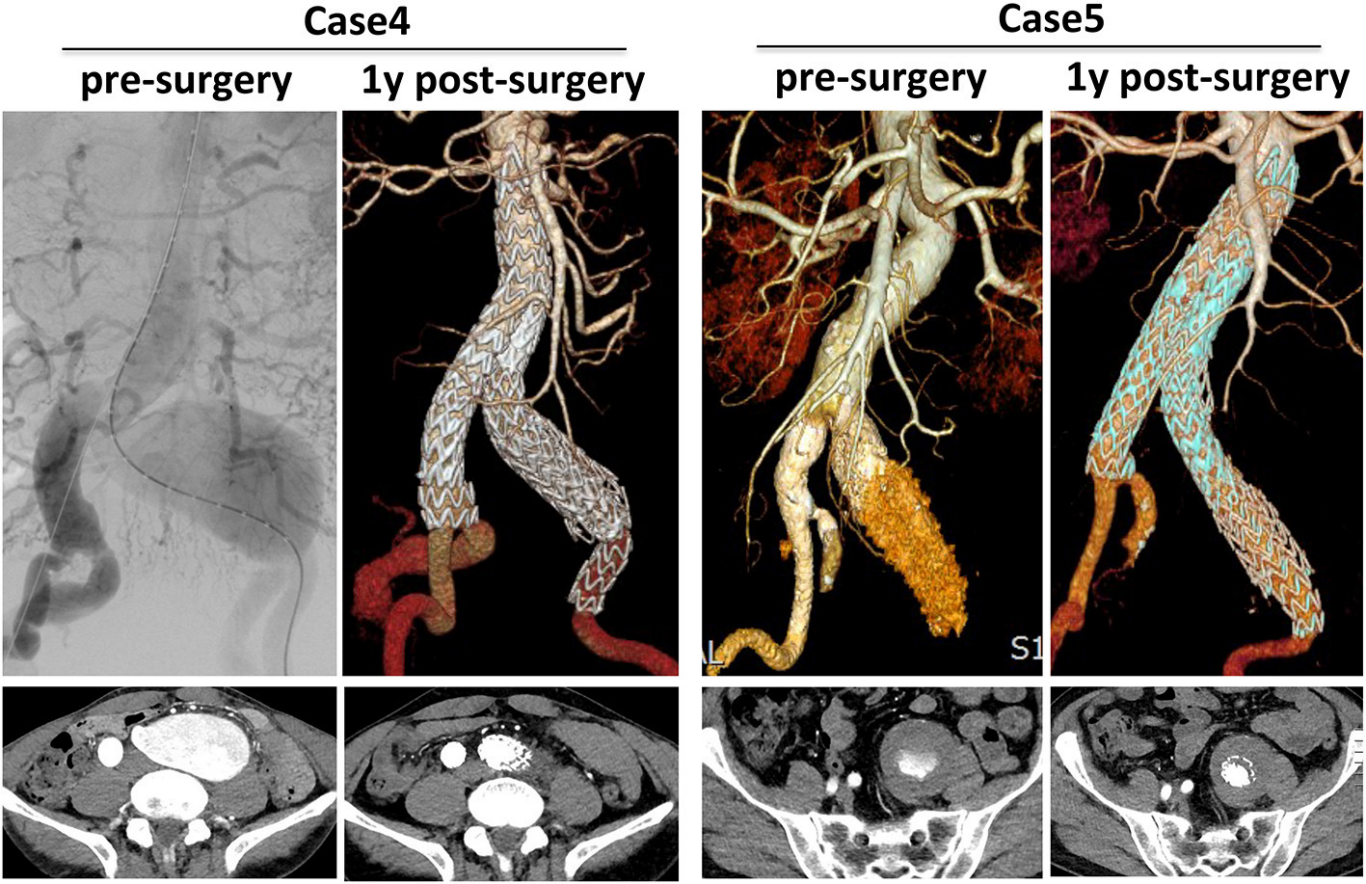
Typical cases of CST for isolated iliac aneurysms. 3D CTA pre-surgery and 1-year post-surgery was compared and corresponding transverse section was also presented.

Cases 4 and 5 showed application of CST for isolated CIAAs (Figure 3). Case 4 showed a satisfactory result of sealing a 50.3 mm CIAA sequentially by a 16–24 mm graft, a 28–28 mm iliac limb, and two 16–16 mm iliac limbs, with the first 16–24 skirt limb located at the orifice of CIA and second 28–28 skirt limb located at the distal bifurcation of CIA. The double skirt-technology fully prevented any potential endoleaks to the aortic sac. Case 5 showed a long and huge CIA with 61 mm maximal diameter, which was also excluded by double CST skill, composed by a graft series of a 16–24, a 16–16, another 16–24 and 16–16 mm graft.

**Figure 4:**
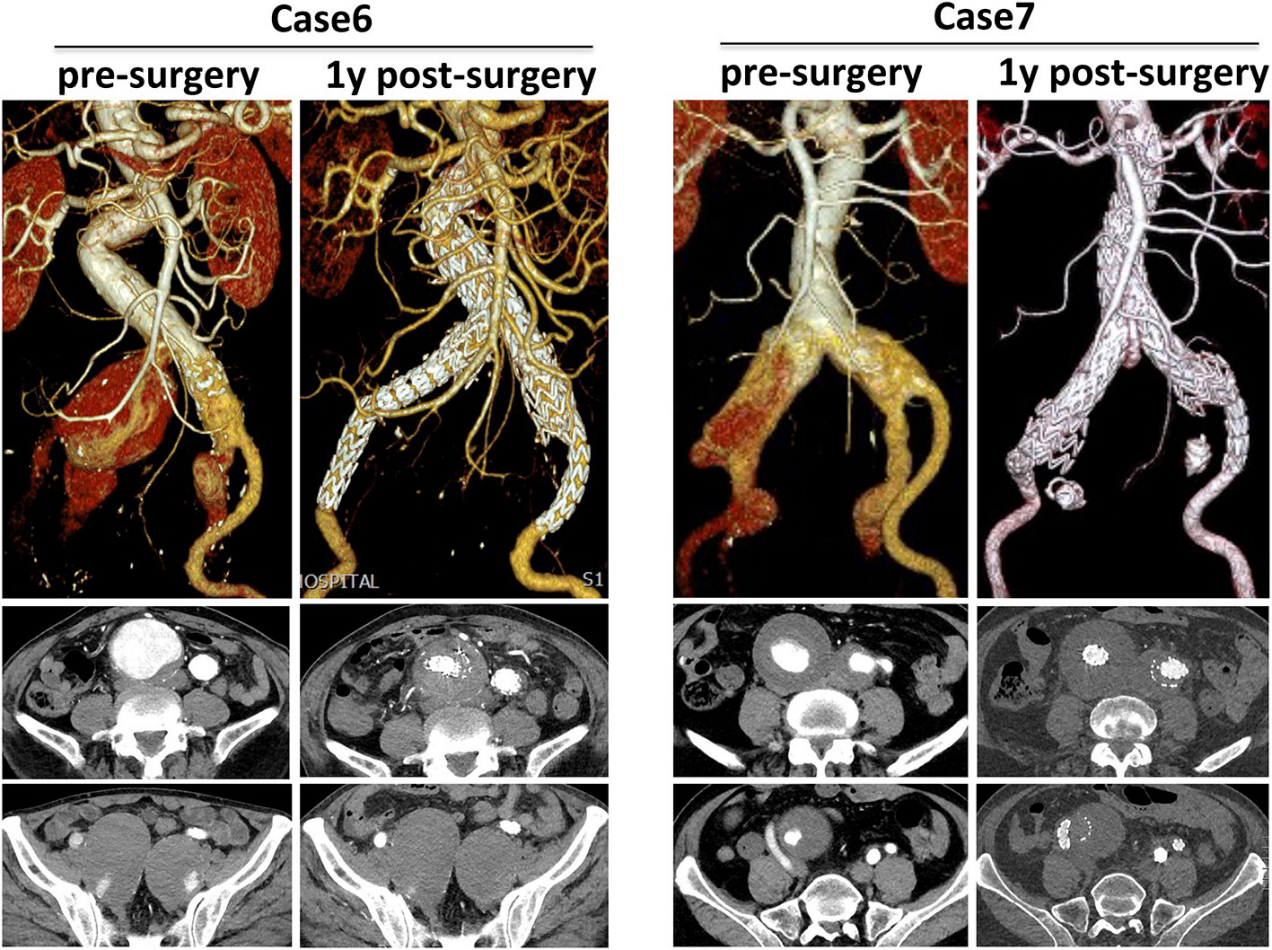
Different strategies in treating IIAA in CST group. 3D CTA pre-surgery and 1-year post-surgery was compared and corresponding transverse section was also presented.

Cases 6 and 7 showed different strategies in treating IIAA in CST group (Figure 4). Both cases showed bilateral CIAA concomitant with IIAAs. For case 6, a series of 28–28, 16–13, 16–13 mm grafts were formed a CST for the right side, and a 16–24 followed with a 16–13 mm graft were used for the left side. Post-surgery and 1-year follow-up results showed complete seal of aneurysm, exclusion of IIA blood flow, no endoleak, and no aneurysm sac enlargement. For case 7, both CIAAs were treated by a 28–28 graft combined with a 16–13 mm iliac limb after coil embolization of the IIA. One-year follow-up showed satisfactory seal of aneurysm and complete embolization of IIA, though light type II endoleak from inferior mesenteric artery existed.

## Discussion

Strategies of treating IIA changed during the development of AAA treatment. At the early stage of AAA open surgery, preservation of at least one IIA was thought to be necessary and bypass of IIA to graft was used both in our center and others (19). Then, with the development of endovasular surgery as first line therapy and more and more data of safety on IIA exclusion, save of IIA was secondary to providing adequate landing zone and complete sealing of aneurysms (11, 13, 15). However, with development of new devices and technologies, preservation of IIA and effort to decrease BC and ED become more and more important. With development of IBDs and improved Quality Control of AAA surgery, a preservation era of IIA is thought coming (20).

But, such new techniques still could be limited by anatomical constraints, technique experience, and availability of grafts. For example, IBDs were not available in China until March 2021. After full evaluation of patient condition, activity level, length, tortuosity and thrombus burden of iliac arteries, and cost, exclusion of IIA was still an indispensable technology in selected cases. And here we reported a novel common iliac artery skirt technology for feasible-and-effective IIA exclusion.

Based on retrospective analysis of data from 39 CST and 26 IIAE patients, our experience showed both groups had comparable primary end points and CST had better secondary end points, including significant short surgery time and hospital stay, lower in-hospital and 1-year stent MAEs, comparable mortality, and stent MAEs for all-cohort follow-up. Results of CST showed clear advantages over IIAE in excluding IIA and at the same time preventing the occurrence of type II endoleaks. Actually, the use of a flared grafts in excluding IIA with or without embolization also published in case reports, either with an upside-down or reversed bell-bottom technique (21–24). But, either a manually unsheathed-and-mounted procedure or a contralateral femoral access and crossover process is needed, respectively, which are not necessary for our CST.

Advantages of CST come from its simplicity and which results in short-time surgery and anesthesia, and less endovascular procedures. Ischemia of pelvic is the main concern and complication for exclusion of hypogastric artery. Literature reports of BC rate were 36.5% for bilateral IIA interruption and 27.2% for unilateral IIA interruption (25), and the erectile dysfunction rate was 12.7%. In our study, the in-hospital BC rate was 20.5% for CST vs. 46.2% for IIAE, *p* = 0.053 and the ED rate was 10.3% for CST vs. 23.1% for IIAE, *p* = 0.352. The direct coverage of orifice of IIA was suggested to have lower rate of BC than embolization of IIA in our study, and evidence was also reported in published data (11, 26–28). Nevertheless, the high preserve rate of contralateral IIA in CST should also not be ignored, thought not statistically significant. After 1 year, about one third BC was relived in both groups (CST 37.5% vs. IIAE 33.3%, *p* = 1.000), and which all happened on patients who had a preserved contralateral IAA. Thus, the save of at least one IIA should be guaranteed as long as possible, as per guidelines (1, 29). And as both CST and IIAE have high and comparable technique successful rate and exclusion of IIA in this study by our group, no difference of mortality and stent MAEs in long term follow-up was found.

Whereas, the critical size for isolated IIAAs treatment is considered to be 3–4 cm (30–32), whether its benefit to treat smaller IIAAs in patients undergoing EVAR for aorta-common iliac aneurysm to avoid the need for secondary interventions is still not clear. And also, for CST group, concerns may remain on risk of future IIAA enlargement by only covering the orifice of IAA. In our study, by subgroup analysis of 14 patents concomitant with IIA dilation in CST group without IIAE and the 7 CST+IIAE patients, no difference was found in mortality, stent MAEs, sac dilation, or reintervetion between two groups. However, considering the challenging of secondary interventions for IIAAs after CST, we recommend that CST plus IIA embolization or other IIAAs procedures should be applied for IIAAs > 35 mm (33).

Overall, the common-iliac-artery skirt technology is simple, since it does not require neither particular endovascular expertise of the operator nor peculiar devices, which has decisive advantage in ruptured patients as a fast and life-saving treatment option. Besides, as the application of CST only restricted by the diameter of CIA bifurcation and the 10% oversize was not strictly needed for the skirt limb, CST was available for majority cases by using available flared iliac limbs and cuffs (maximal 36 mm). Comparing to IIAE, our experience showed CST had advantages in reducing surgery time, hospital stay, stent related MAEs, and BC rates. Satisfying safety and sealing of CIAAs without type II endoleak was also obtained.

Unilateral application of CST plus a patency or preserved contralateral IIA could be a satisfactory solution for most challenge iliac artery aneurysms. Nevertheless, larger sample size and long-term follow-up is warranted to assess the durability of the technology and absence of re-interventions.

As a retrospectively study, limitations of this study may include potential selection bias of patients for surgical method. However, comparable results of majority risk factors were found between groups.

## Conclusion

In selected cases, the CST can be used for the complete exclusion of challenge iliac artery aneurysms and preventing type II endoleak from the IIA, especially for those who has advanced age, or contralateral health IIA, or cannot tolerate long-time surgery and is limited in activities, or when preservation of IIA does more harm than good or is not practical.

## Data Availability Statement

The raw data supporting the conclusions of this article will be made available by the authors, without undue reservation.

## Ethics Statement

The studies involving human participants were reviewed and approved by Ethics Committee of the 2nd Xiangya Hospital, Central South University. The patients/participants provided their written informed consent to participate in this study. Written informed consent was obtained from the individual(s) for the publication of any potentially identifiable images or data included in this article.

## Author Contributions

CS, LW, QL, ML, HH, and XL contributed to conception and design of the study. LW, YS, JQ, TW, CY, MW, and JL collected data and organized the database. HW and LS performed the statistical analysis. LW wrote the first draft of the manuscript. All authors contributed to manuscript revision, read, and approved the submitted version.

## Funding

This study was supported with National Natural Science Foundation of China for LW (81900423), CS (81870345), and JL (81800400) and Natural Science Foundation of Hunan Province for LW (2020JJ5836) and JL (2019JJ50851).

## Conflict of Interest

The authors declare that the research was conducted in the absence of any commercial or financial relationships that could be construed as a potential conflict of interest.

## Publisher's Note

All claims expressed in this article are solely those of the authors and do not necessarily represent those of their affiliated organizations, or those of the publisher, the editors and the reviewers. Any product that may be evaluated in this article, or claim that may be made by its manufacturer, is not guaranteed or endorsed by the publisher.
